# Identifying factors associated with adolescents’ Intention for childbirth

**DOI:** 10.34763/jmotherandchild.20222601.d-22-00022

**Published:** 2023-02-22

**Authors:** Dimitra Varnakioti, Antigoni Sarantaki, Kleanthi Gourounti, Aikaterini Lykeridou

**Affiliations:** Faculty of Health and Caring Sciences, Midwifery Department, University of West Attica, Athens, Greece

**Keywords:** Adolescents, Attitudes, Vaginal birth, Caesarean section, Theory of Planned Behavior, Educational interventions

## Abstract

**Background:**

Around the world, caesarean section rates have steadily increased over the past few decades. The World Health Organization (WHO) guidelines on nonclinical interventions to reduce caesarean section rates emphasize educational interventions and support programs.

**Material and methods:**

In this study, we have determined factors associated with adolescents’ intention regarding childbirth options using the Theory of Planned Behavior (TPB). The sample was comprised of 480 high school students in Greece who were invited to complete a questionnaire consisting of three sections: a section on sociodemographic data; a section featuring the Adolescents’ Intentions towards Birth Options (AIBO) scale, a recently developed instrument that accesses attitudes and intentions regarding vaginal birth and caesarean section; and a section detailing participants’ awareness regarding reproduction and birth.

**Results:**

Multiple logistic regression found that participants’ impressions of vaginal birth and the TPB constructs were significantly associated with intention towards caesarean section. In particular, participants with a negative impression of vaginal birth had a 2.20-fold higher probability of reporting their preference for caesarean section, compared to participants with neither a negative nor a positive impression. Furthermore, participants with higher scores on the “Attitudes towards vaginal birth,” “Subjective norms,” and “Perceived behavior control over vaginal birth” subscales had a significantly lower probability of reporting preference for caesarean section.

**Conclusion:**

Our study demonstrates the effectiveness of the TPB to identify factors that influence adolescents’ preference for childbirth. We highlight the necessity to implement nonclinical interventions to reduce the preference for caesarean section, providing evidence for developing school-based educational programs for a timely and consistency implementation.

## Introduction

Around the world, caesarean section (CS) rates have steadily increased over recent decades [1]. Caesarean sections can effectively prevent maternal and newborn mortality when it is medically indicated. However, there is evidence that they are associated with short- and long-term risks for the woman, the child, and future pregnancies, in addition to considering public healthcare costs [2].

The rise in caesarean section rates can be explained through a complex multivariate analysis of the following contributing factors: changes regarding both the distinguishing characteristics of the mother (with pregnancies being increased in elderly nulliparous women) and professional practice styles; personalized medicine instead of team obstetric care; increased malpractice pressure (defensive medicine); and economic, organizational, social, and cultural factors [3,4,5,6]. A recent study indicates that in Greece, during the financial and COVID-19 pandemic crises, most women continue to give birth by CS, which leads to a major public health problem with economical, ethical, and humanitarian implications [7].

While the debate for interventional approaches is ongoing, the World Health Organization (WHO) guidelines on nonclinical interventions attempt to provide evidence-based recommendations specifically designed to reduce caesarean section rates. The WHO has emphasized educational interventions and support programs, such as childbirth training workshops that include sessions about childbirth fear and pain, non-pharmacological pain-relief methods, and the advantages and disadvantages of caesarean section and vaginal delivery [8].

### The Planned Behavior Theory

Social cognitive theory models indicating that attitudes directly affect behaviors are currently being tested to predict the preferred birth approaches [9, 10, 11, 12]. Ajzen’s theory of planned behavior (TPB) has been used successfully to explain and predict behavior in a multitude of behavioral domains: from physical activity to drug use, recycling, choice of travel mode, safer sex, consumer behavior, technology adoption, protection of privacy, and more. According to the TPB, behavioral intention is determined by three constructs: attitudes (positive or negative assessment, knowledge, emotions) towards the behavior, subjective norms (the influence of significant people, e.g. relatives and health professionals) and perceived behavior control (derived from Bandura’s concept of self-efficacy, referring to the perception of the amount of control over behavior implementation) [13].

In this study, we have determined the factors associated with young adolescents’ preference and intention regarding vaginal birth and caesarean section using the TPB. In order to estimate adolescents’ awareness in regard to reproduction and birth, we also consulted various sources of information on these issues.

## Material and methods

A cross-sectional study was conducted in 14 vocational high schools in Greece, with ten school units being in the Athens area and four school units being in the countryside. Data was collected via a questionnaire between October 2019 and April 2021. The study protocol was reviewed and approved by the Ιnstitute of Educational Policy, which is the scientific agency that provides support to the Minister of Education and Religious Affairs on issues regarding secondary education (No Φ12/124243/Δ4 1/8/2019). Eligible participants were ensured confidentiality and anonymity in their responses. Written consent was received from all participants and their parents before filling in the questionnaire. Due to the specialized nature of the project, we provided instructions in every school unit and additional clarifications were available in classrooms for each of the students during questionnaire completion.

The sample was comprised of 480 students (54.2% girls) aged 14–17 years, with a mean age of 15.5 years (SD = 0.6). The sample characteristics are presented in Table 1. The majority of the sample (97.7%) was born in Greece. More than half of the participants had parents who had completed high school and 55.8% had chosen to study health sciences.

**Table 1:** Sample characteristics

	*N* (%)
Age, mean (SD)	15.5 (0.6)
Gender	
Boys	209 (43.5)
Girls	260 (54.2)
Prefer not to say	11 (2.3)
Born in Greece	469 (97.7)
Father’s highest level of education	
Primary school completed	69 (14.4)
High school completed	268 (55.8)
Technical Institute (VTI) School-Vocational Training	78 (16.3)
University degree	53 (11)
Postgraduate degree	8 (1.7)
Doctoral diploma (PhD)	4 (0.8)
Mother’s highest level of education	
Primary school completed	44 (9.2)
High school completed	251 (52.3)
Technical Institute (VTI) School-Vocational Training	92 (19.2)
University degree	74 (15.4)
Postgraduate degree	14 (2.9)
Doctoral diploma (PhD)	5 (1.0)
Field of study	
Agriculture and Environment	8 (1.7)
Administration and Economics	42 (8.8)
Structured Design Environment and Architectural	9 (1.9)
Applied Arts	15 (3.2)
Electronics and Automation	19 (4.0)
Engineering	55 (11.6)
Shipping and Maritime Studies	16 (3.4)
Computing Studies	46 (9.7)
Health Sciences	265 (55.8)

### Phase I

This phase was designed for scale development. In order to access students’ preference and intention towards vaginal birth and caesarean section, a multidimensional instrument was developed to access the three main dimensions of the Theory of Planned Behavior (attitudes, subjective norms and perceived behavioral control).

Through an extensive literature review, we identified data that examined attitudes towards birth decisions among men and women (pregnant and nonpregnant). A focus group of six experts from different fields evaluated the face validity and the content validity index (CVI) of the developed scale.

The scale was prepared in Greek, and the psychometric evaluation assessed reliability, exploring the factor structure of the instrument and the internal consistency by using Cronbach’s alpha coefficient, and assessed the stability of the tool by evaluating the test-retest reliability via intraclass correlation coefficient (ICC) and the discriminant and construct validity by using known groups method testing.

The test-retest reliability of the scale was calculated for 30 out of 480 students who agreed to repeat the scale two weeks after the first administration. Significant agreement in all factors emerged from the test-retest assessment.

The results of the exploratory factor analysis suggested that discriminative capacity existed among the initial items and that a five-factor solution was the most appropriate. The factors were interpreted by the authors, and the attitude constructs were labeled as follows in accordance with the TPB: “Cognitive component of vaginal birth” for knowledge and beliefs about vaginal birth, “Affective component of vaginal birth” for emotions regarding vaginal birth, “Cognitive component of caesarean section” for knowledge and beliefs for caesarean section, “Subjective norms-significant others” for family’s and health professionals’ beliefs about delivery mode, and “Perceived behavior control” for participants’ perception of their control over a hypothetic vaginal birth. Every question was answered by a 5-item Likert scale ranging from “strongly disagree = 1” to “strongly agree = 5”. Lower scores indicated negative attitudes and higher scores indicated positive attitudes. The scale was named “Adolescents’ Intentions towards Birth Options Scale” (AIBOS) (Appendix).

The overall Cronbach’s alpha coefficient was 0.76, indicating acceptable internal consistency of the scale.

Discriminant construct validity was evaluated by analysing the association between the factors of the questionnaire and gender, field of study, and preference for a specific type of labor using students’ t-tests. Statistical significance was set at p<0.05 and analyses were conducted using IBM SPSS Statistics for Windows, Version 24.0 (Armonk, NY: IBM Corp). It was found that girls had significantly greater mean scores in all factors, except in “Knowledge on caesarian section,” compared to boys. Teenagers who would study health sciences in the future had significantly greater scores in “Knowledge on vaginal labor,” “Affective attitude,” and “Subjective norms,” and significantly lower scores in “Knowledge on caesarian section” in comparison to teenagers who would study non-medical sciences. Moreover, teenagers who would prefer to have a vaginal birth for themselves or their partner had significantly greater scores in “Knowledge on vaginal labor,” “Affective attitude,” “Subjective norms,” and “Perceived behavior control,” and significantly lower scores in “Knowledge on caesarian section,” in comparison to teenagers who would prefer to have a caesarian section [14].

### Phase II

#### Measures

All students of the first grade were invited to complete a questionnaire consisting of three sections. In section A, sociodemographic variables such as age, gender, parents’ level of education and students’ field of study were assessed. In section Β, attitudes, subjective norms, and perceived behavior control were examined via the AIBO scale. Section C, in order to survey students’ awareness in regard to birth and reproductive health, assessed sources of information, impressions towards pregnancy and birth, and students’ reported knowledge gaps about the afore mentioned issues.

It is worth mentioning that the instructions requested the participants to keep in mind their role: a girl who will manage a future healthy pregnancy, or a boy who will support a pregnant woman.

#### Statistical analysis

Quantitative variables were expressed as means and standard deviations, whereas qualitative variables were expressed as absolute and relative frequencies. For comparison of proportions, chi-square and Fisher exact tests were used. Independently sampled students’ *t*-tests were used for comparison of mean values between the two groups. Logistic regression analysis was used to find independent factors associated with the intention to select caesarean section. Adjusted odds ratio (OR) and 95% confidence interval (95% CI) were computed from the results of the logistic regression analyses. The Hosmer–Lemeshow statistic was used to check the goodness of fit of the logistic regression models. The betas and standard errors are not provided; since it’ was a logistic and not a linear regression analysis, the betas are not interpretable and could not directly provide likelihood comparisons. Instead, we have provided odds ratios along with a 95% confidence interval. With odds ratios (Exp(b)) we can directly understand the effect of the independent variables. All reported p-values are two-tailed. Statistical significance was set at p < 0.05 and analyses were conducted using IBM SPSS Statistics for Windows, Version 22.0 (Armonk, NY: IBM Corp).

## Results

Participants’ intentions towards vaginal birth and their associations with sources of information are presented in Table 2. Almost nine out of ten (89.0%) of the students could imagine having children at some point in the future and 78.5% would prefer a vaginal birth for them or their partner. The most common source of information on vaginal birth was “experiences/stories of family members” (37.7%). Impressions of the sample regarding vaginal birth were positive for 37.9%, but both negative and positive for 49.0%. The majority of students in the sample (54.1%) would like to be more informed in all subject areas of pregnancy and birth. The mean scores on the five subscales of the TPB were as follows: “Cognitive component of vaginal birth”: 3.53 (SD = 0.61); “Cognitive component of caesarean section”: 3.00 (SD = 0.64); “Affective component of vaginal birth”: 3.78 (SD = 0.63); “Subjective norms”: 4.01 (SD = 1.13); and “Perceived behavior control”: 2.91 (SD = 0.92).

**Table 2:** Participants’ intention towards vaginal birth and the sources of information

	*N* (%)
Can you imagine yourself having children sometime in the future?	427 (89.0)
Your attitude towards pregnancy and vaginal birth was shaped mainly from:	
Visual means	57 (11.9)
Written means	8 (1.7)
Friends’ experiences/stories	62 (12.9)
Experiences/stories of family members	181 (37.7)
School	23 (4.8)
Other	8 (1.7)
All the above	141 (29.4)
From the medium from which you were informed about vaginal birth, your impressions were, on average:	
Positive	182 (37.9)
Negative	24 (5.0)
Both	235 (49.0)
Neither positive nor negative	39 (8.1)
Subjects you wish to be more informed about:	
The procedure of pregnancy	49 (10.2)
Promotion of a healthy pregnancy (diet, way of living)	38 (7.9)
The procedure of labor	41 (8.5)
Available services of healthy reproduction	13 (2.7)
What could go wrong during pregnancy and labor	57 (11.9)
How both partners could be included in the experi- ence of birth	23 (4.8)
All the above	259 (54.1)
Assuming you could choose the type of birth for your baby, would you prefer it to be a:	
Vaginal birth	377 (78.5)
Caesarean section delivery	103 (21.5)

Participants who could imagine having children at some point in the future tended to prefer vaginal birth by a significantly larger percentage than participants who did not imagine having children at some point in the future (Table 3). Furthermore, participants’ intention to have a specific mode of delivery was significantly associated with their impression of vaginal birth. Specifically, a significantly higher percentage of participants who had negative impressions intended to give birth by caesarean section (Table 4).

**Table 3:** Participants’ intention for vaginal birth or caesarean section associated with their characteristics, sources of information and the theory of planned behavior (TPB) constructs

		Assuming you could choose the type of birth for your baby, would you prefer it to be a:		
		Vaginal birth	Caesarean section delivery	
		*N* (%)	*N* (%)	P
Gender	Boys	168 (80.4)	41 (19.6)	0.419+
	Girls	201 (77.3)	59 (22.7)	
Age, mean (SD)		15.5 (0.6)	15.5 (0.6)	0.592‡
Father’s highest level of education	Primary school completed	53 (76.8)	16 (23.2)	0.549+
	High school completed	215 (80.2)	53 (19.8)	
	Technical School-Vocational Training Institute (VTI)	62 (79.5)	16 (20.5)	
	University degree/postgraduate degree/doctoral	47 (72.3)	18 (27.7)	
	diploma (PhD)			
Mother’s highest level of education	Primary school completed	33 (75.0)	11 (25.0)	0.898+
	High school completed	200 (79.7)	51 (20.3)	
	Technical School-Vocational Training Institute (VTI)	72 (78.3)	20 (21.7)	
	University degree/postgraduate degree/doctoral diploma (PhD)	72 (77.4)	21 (22.6)	
Born in Greece	Yes	367 (78.3)	102 (21.7)	0.470++
	No	10 (90.9)	1 (9.1)	
Field of study	Other	159 (75.7)	51 (24.3)	0.184+
	Health Sciences	214 (80.8)	51 (19.2)	
Can you imagine yourself having	No	34 (64.2)	19 (35.8)	**0.007+**
children sometime in the future?	Yes	343 (80.3)	84 (19.7)	
Your attitude towards pregnancy and vaginal birth was shaped mainly	Visual means	42 (73.7)	15 (26.3)	0.886++
from:	Written means	6 (75.0)	2 (25.0)	
	Friends’ experiences/stories	48 (77.4)	14 (22.6)	
	Experiences/stories of family members	142 (78.5)	39 (21.5)	
	School	20 (87.0)	3 (13.0)	
	Other	6 (75.0)	2 (25.0)	
	All the above	113 (80.1)	28 (19.9)	
From the medium from which you	Positive	163 (89.6)	19 (10.4)	**<0.001+**
were informed about the vaginal birth, your impressions were, on	Negative	9 (37.5)	15 (62.5)	
average:	Both	172 (73.2)	63 (26.8)	
	Neither positive nor negative	33 (84.6)	6 (15.4)	
Subjects you wish to be more informed about:	The procedure of pregnancy	41 (83.7)	8 (16.3)	0.482+
	Promotion of a healthy pregnancy (diet, way of living)	34 (89.5)	4 (10.5)	
	The procedure of labor	32 (78.0)	9 (22.0)	
	Available services of healthy reproduction	11 (84.6)	2 (15.4)	
	What could go wrong during pregnancy and labor	41 (71.9)	16 (28.1)	
	How both partners could be included in the experi- ence of birth	18 (78.3)	5 (21.7)	
	All the above	200 (77.2)	59 (22.8)	
“Cognitive component of vaginal birth” subscale, mean (SD)		3.62 (0.59)	3.22 (0.57)	<0.001‡
“Cognitive component of caesarean section” subscale, mean (SD)		2.91 (0.63)	3.36 (0.54)	<0.001‡
“Affective component of vaginal birth” subscale, mean (SD)		3.82 (0.63)	3.61 (0.64)	0.002‡
“Subjective norms” subscale, mean (SD)		4.29 (0.88)	3.00 (1.36)	<0.001‡
“Perceived behavior control” subscale, mean (SD)		3.2 (0.86)	2,68 (1.02)	<0.001‡

+Pearson’s chi-square test; ++Fisher’s exact test; ‡Student’s *t*-test.

**Table 4:** Multivariate logistic regression analysis results, with the intention for caesarean section as dependent variable

		OR (95% CI) +	P
Gender	Boys (reference)		
	Girls	3.97 (0.82─19.22)	0.087
Age		1.10 (0.66─1.82)	0.720
Father’s highest level of education	Primary school completed (reference)		
	High school completed	0.90 (0.32─2.47)	0.830
	Technical School-Vocational Training Institute (VTI)	0.75 (0.23─2.49)	0.642
	University degree/postgraduate degree/doctoral diploma (PhD)	1.10 (0.33─3.74)	0.875
Mother’s highest level of education	Primary school completed (reference)		
	High school completed	1.42 (0.45─4.49)	0.555
	Technical School-Vocational Training Institute (VTI)	1.14 (0.33─4.02)	0.834
	University degree/postgraduate degree/doctoral diploma (PhD)	1.04 (0.29─3.73)	0.957
Born in Greece	Yes (reference)		
	No	0.25 (0.02─2.81)	0.263
Field of study	Other (reference)		
	Health Sciences	0.68 (0.35─1.33)	0.257
Can you imagine yourself having children some- time in the future?	No (reference) Yes	0.90 (0.37─2.21)	0.816
From the medium from which you were informed about on average: the vaginal birth, your impressions were,	Neither positive nor negative (reference) Positive Negative	1.26 (0.35─4.49) 2.20 (1.06─4.54)	0.725 0.034
	Both	2.58 (0.79─8.42)	0.116
“Cognitive component of vaginal birth” subscale		0.36 (0.20─0.64)	<0.001
“Cognitive component of caesarean section” subscale		2.80 (1.59─4.91)	<0.001
“Affective component of vaginal birth” subscale		0.55 (0.33─0.92)	0.022
“Subjective norms” subscale		0.45 (0.35─0.59)	<0.001
“Perceived behavior control” subscale		0.49 (0.35─0.69)	<0.001

+Odds ratio (95% confidence interval) Note: p=0.785 from Hosmer–Lemeshow statistic

Multiple logistic regression found that participants’ impressions of vaginal birth and all the TPB constructs were significantly associated with intention towards caesarean section (Table 4). More specifically, participants with a negative impression of vaginal birth had a 2.20-fold higher probability of selecting caesarean section compared to participants with neither negative nor positive impressions. Also, higher scores on the “Cognitive component of vaginal birth,” “Affective component of vaginal birth,” “Subjective norms,” and “Perceived behavior control” subscales were significantly associated with a lower probability of selecting caesarean section. A higher score on the “cognitive component of caesarean section” subscale was also significantly associated with a higher probability of selecting caesarean section.

## Discussion

Birth-related attitudes during pregnancy are frequently considered and analysed. Strategies to raise awareness about the benefits of vaginal birth, to promote women’s confidence in their ability to deliver naturally, are being updated. For young populations, although research indicates that developing and supporting evidence-based education programs is vital, the availability of data regarding adolescents’ attitudes towards birth options is scarce [15, 16].

In our study, most participants would prefer a vaginal birth for them or their partner in a hypothetical healthy future pregnancy. In accordance with this, studies in similar populations indicate that, regardless of the recorded rise in the rate of caesarean sections, young students who are introduced into a medicalized birth culture report a vaginal birth preference for a healthy future pregnancy [17, 18, 19]. However, considering that the mean age of the sample in our study was 15.5 years, the intention for a caesarean delivery was already 21.1%, a percentage remarkably higher than the justified 10–15% of medically indicated caesarean sections according to the WHO. The most common source of information on vaginal birth was “experiences/stories of participants’ family members” and additional studies have indicated the strong influence that family and the peer network have on birth preferences in young adults [20, 21]. Participants’ intentions for a specific mode of delivery were significantly associated with their impression of vaginal labor. Students who had negative impressions had a significantly higher likelihood of intending to give birth by caesarean section. A similar outcome was reported in a survey study that assessed impressions of childbirth in a student population in Northwest England. The authors supported that firsthand observations of birth, especially positive experiences, had implications for salutary outcomes, whereas negative or conflicting perceptions of vicarious experiences were associated with increased levels of fear over childbirth [22].

Regarding the factors on the AIBO scale related to participants’ preference for mode of birth, we found that all subscales were significantly associated with the intention for caesarean section. More specifically, participants with higher scores on the “Cognitive component of vaginal birth,” “Affective component of vaginal birth,” “Subjective norms,” and “Perceived behavior control” subscales had a significantly lower probability of selecting caesarean section. In contrast, a higher score on the “Cognitive component of caesarean section” subscale was significantly associated with a higher probability of selecting caesarean section. This outcome is consistent with the effectiveness of the TPB to predict and explain the correlation between factors and intention.

Taking into consideration the significant association between intention and the AIBO scale constructs, we indicate the necessity to modify the negative attitudes towards vaginal birth and educate young populations about the benefits and risks of different modes of delivery. In line with recent WHO guidelines for nonclinical interventions to reduce caesarean section rates, such as childbirth preparation workshops, we suggest introducing health and reproduction education programs into the school curricula in primary schools and most certainly in high schools. The fact that only 13% of our sample reported school-based education programs as a source of information in birth issues indicates the educational inadequacy in Greece. Furthermore, our study showed that significant others, obstetricians, and midwifes can affect adolescents’ intention towards a particular mode of delivery; thus, they could, and should, be involved with providing information from a more scientific perspective.

Perceived behavior control over childbirth seems to be another factor related to delivery preference. A recent study suggests that increased self-efficacy in childbirth is associated with a wide variety of improved perinatal outcomes and that self-efficacy is a psychosocial factor that can be modified through various efficacy-enhancing interventions [23]. This outcome emphasizes the power of prenatal education for improving individuals’ confidence in coping with a vaginal delivery.

**Table 5:** Participants’ preference of birth, associated with their gender and the theory of planned behavior (TPB) constructs

			Boys					Girls				
			Assuming you could choose the type of birth for your baby, would you prefer it to be a:					Assuming you could choose the type of birth for your baby, would you prefer it to be a:				
	Total sample		Vaginal birth		Caesarean section			Vaginal birth		Caesarean section		
	Mean	SD	Mean	SD	Mean	SD	P*	Mean	SD	Mean	SD	P
“Cognitive component of vaginal birth” subscale	3.53	.61	3.53	.55	3.17	.56	<0.001	3.71	.61	3.24	.58	<0.001
“Cognitive component of caesarean section” subscale	3.00	.64	2.98	.55	3.38	.60	0.001	2.84	.70	3.33	.51	<0.001
“Affective component of vaginal birth” subscale	3.78	.63	3.61	.64	3.35	.68	0.025	4.00	.55	3.78	.57	0.007
“Subjective norms” subscale	4.01	1.13	4.25	.88	2.37	1.08	<0.001	4.30	.88	3.45	1.36	<0.001
“Perceived behavior control” subscale	2.91	.92	2.67	.73	2.84	.90	0.202	2.89	.94	3.61	1.00	<0.001

Student’s t-test

A more recent study examined stakeholders’ views on the barriers and facilitators of nonclinical interventions to reduce unnecessary caesarean sections, targeted at organizations, facilities, and systems. The authors reported that these interventions were strongly mediated by organizational power differentials and stakeholder commitment. The study also indicated that the barriers are greatest where the implementation plans contradict both system and cultural norms [24].

### Limitations

This study’s results need to be interpreted by taking into consideration its limitations. First, convenience sampling was used, although this was not planned. The research protocol had anticipated the participation of schools from various geographical departments; however, the emergence of the COVID-19 pandemic made all school communities wary of face-to-face activities. Additionally, the sample of students was drawn only from vocational high schools due to the limitations of the responsible National Educational Institute stating that any surveys should be integrated in the relevant courses. It is also worth mentioning that, ideally, the researchers would have wanted to have collected data about the emotional state of the participants, but this was prevented due to the low average age of the sample.

Thus, this study may have introduced selection bias and produced a non-representative sample of students; therefore, the results may not be generalizable to other populations.

## Conclusions

In conclusion, in our study, we assessed sources of information and TPB constructs to identify factors influencing adolescents’ preference and intention towards vaginal birth and caesarean sections. To the best of our knowledge, this is the first such study in Greece with such a low average age of participants, and it indicated that adolescents’ birth preferences were significantly associated with sources of information, attitudes towards the delivery mode, subjective norms, and childbirth perceived behavior control. The findings highlight the necessity to implement nonclinical interventions to reduce the preference for caesarean section, providing evidence for developing school-based educational programs. We propose that these interventions should be implemented both consistently and in a timely manner. Our study also purports that the rise in caesarean section rates should be approached through a multivariable model that involves individuals, families, educational systems, health professionals, and communities.

## Appendix

**The Adolescents’ Intentions towards Birth Options Scale**

Scale items

Normal or vaginal birth is a completely natural process that occurs at the end of a pregnancy and the baby is delivered vaginally

Women give birth mainly by normal childbirth and caesarean sections in a population should not be more than a small percentage of all births

Vaginal birth is safer for the mother

Vaginal birth is safer for the baby

After a vaginal birth the recovery time is faster and the postpartum pain less

Normal childbirth activates the early bonding of mother-newborn and increases the success of breastfeeding

A caesarean section delivery is a surgical procedure required only for medically indicated reasons (the safety of the mother or the baby)

Caesarean section could be seen as a means to avoid the pain of a vaginal birth

Caesarean section is safer for the mother

Caesarean section is safer for the baby

Caesarean section could be suggested to provide the convenience to schedule the time of childbirth

Vaginal birth is generally unpredictable

I see vaginal birth as an outdated method of childbirth

I believe that significant for me people (family. relatives) would prefer for my delivery (my partner’s delivery) a vaginal delivery

I believe that health care providers (obstetricians. midwives) would prefer for my delivery (my partner’s delivery) a vaginal delivery

I think that I (my partner) will have self-control and will confidently handle the process and the pain of a childbirth

I think that labor pain will be too intense. I am afraid that I (my partner) might panic and not know what to do during labor and birth
